# Supplementary material for: A Newly Established Murine Cell Line as a Model for Hepatocellular Cancer in Non-Alcoholic Steatohepatitis
Source: Int J Mol Sci. 2019 Nov 12;20(22):5658. doi: 10.3390/ijms20225658 (PMC6888677; doi:10.3390/ijms20225658)
Supplement: Supplementary file 1 [file ijms-20-05658-s001.zip › ijms-635656-supplementary-XML/ijms-635656-supplementary-Figures S1-S3.pdf]

a

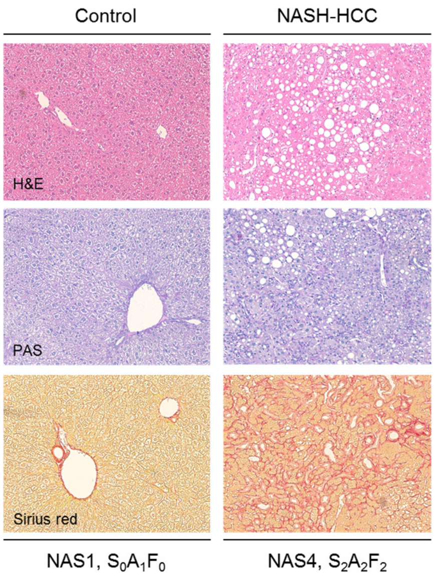

b

Characteristics	Human	N-HCC25 animal model
Weight gain	+	+
Glucose intolerance	+	+
Steatosis	+	+
Lobular Inflammation	+	+
Hepatocyte ballooning	+	+
Fibrosis	+	+
Cirrhosis	+	-
HCC	+	+

Figure S1. (**a**) Representative histological pictures of livers: Comparison of NASH-HCC mice (treatment with DMBA and western diet) to controls (treatment with DMBA and regular diet). (**b**) Metabolic and histopathological characteristics of human and murine NASH-derived HCC.

a

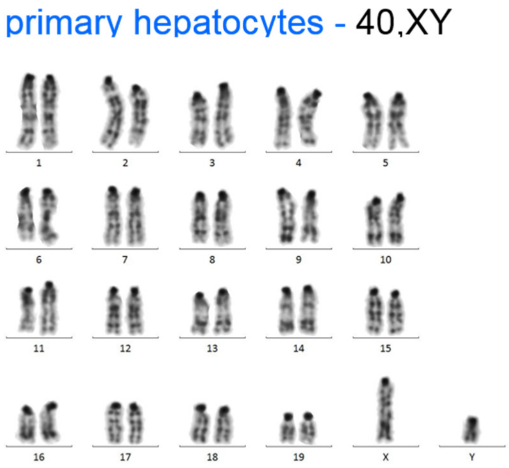

b

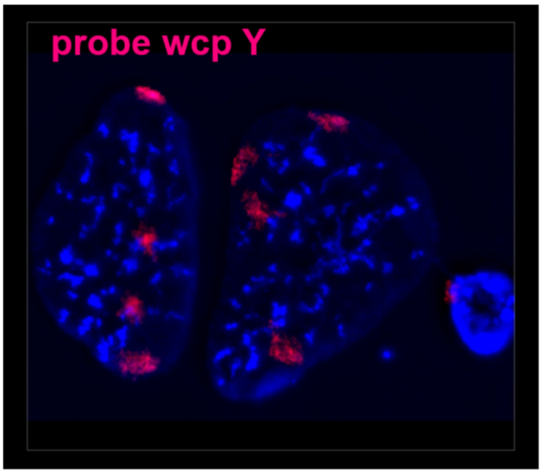

Figure S2: (**a**) Karyogram of wild type primary hepatocytes. (**b**). Representative picture N-HCC25 interphase nuclei with marked amplification of the Y chromosome

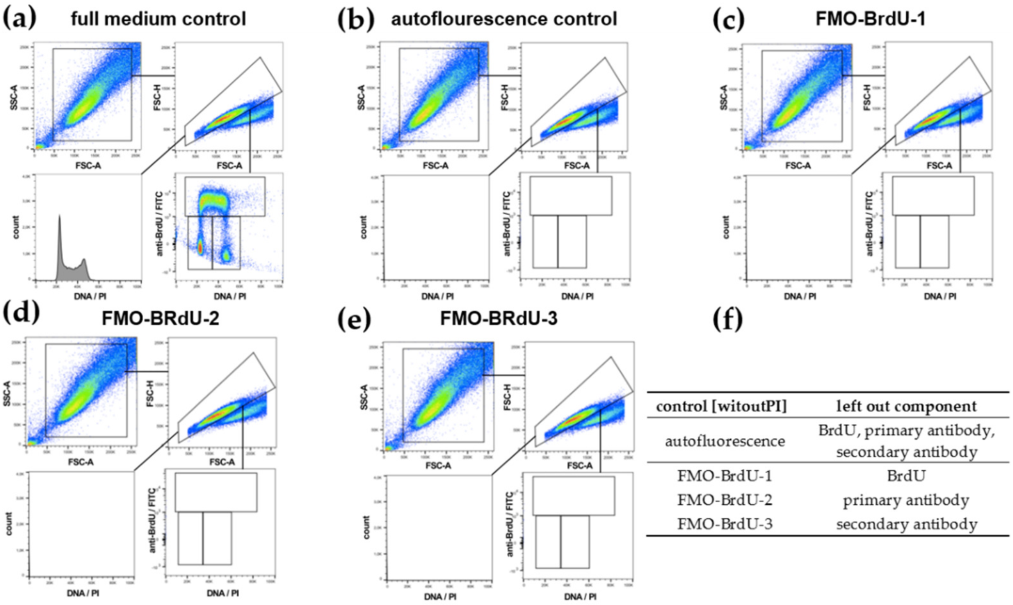

control [witoutPI]	left out component
autofluorescence	BrdU, primary antibody, secondary antibody
FMO-BrdU-1	BrdU
FMO-BrdU-2	primary antibody
FMO-BrdU-3	secondary antibody

Figure S3. Gating strategy and technical controls of flow cytometry with BrdU and PI. The applied gating strategy in flow cytometry experiments is demonstrated in **a** (full medium control with all components). Results of technical controls are shown in **b–e** while the left out components are listed in **f** (FMO = fluorescence minus one).
